# Predicting Pathogenic Variants of Breast Cancer Using Ultrasound-Derived Machine Learning Models

**DOI:** 10.3390/cancers17061019

**Published:** 2025-03-18

**Authors:** Nicoleta Zenovia Antone, Roxana Pintican, Simona Manole, Liviu-Andrei Fodor, Carina Lucaciu, Andrei Roman, Adrian Trifa, Andreea Catana, Carmen Lisencu, Rares Buiga, Catalin Vlad, Patriciu Achimas Cadariu

**Affiliations:** 1 Department of Oncological Surgery and Oncological Gynecology, “Iuliu Hatieganu” University of Medicine and Pharmacy, 400347 Cluj-Napoca, Romaniacatalin.vlad@umfcluj.ro (C.V.);; 2 Breast Cancer Center, Prof. Dr Ion Chiricuta Oncology Institute, 400015 Cluj-Napoca, Romania; adrian.trifa@umft.ro (A.T.); andreea.catana@reginamaria.ro (A.C.); carmen_lisencu@yahoo.com (C.L.); 3 Department of Radiology, Prof. Dr Ion Chiricuta Oncology Institute, 400015 Cluj-Napoca, Romania; pintican.roxana.maria@elearn.umfcluj.ro (R.P.); carinalucaciu@gmail.com (C.L.);; 4 Department of Radiology, “Iuliu Hatieganu” University of Medicine and Pharmacy, 400012 Cluj-Napoca, Romania; 5 Department of Radiology, Niculae Stancioiu Heart Institute, 400001 Cluj-Napoca, Romania; 6 International Institute for the Advanced Studies of Psychotherapy and Applied Mental Health, Babeş-Bolyai University, 400015 Cluj-Napoca, Romania; 7 Department of Clinical Psychology and Psychotherapy, Babeş-Bolyai University, 400015 Cluj-Napoca, Romania; 8 Discipline of Medical Genetics, Center for Research and Innovation in Personalized Medicine of Respiratory Diseases, “Victor Babes” University of Medicine and Pharmacy, 300041 Timisoara, Romania; 9 Center of Expertise on Rare Pulmonary Diseases, Clinical Hospital of Infectious Diseases and Pneumophysiology “Dr. Victor Babes”, 300226 Timisoara, Romania; 10 Department of Genetics, “Iuliu Hatieganu” University of Medicine and Pharmacy, 400012 Cluj-Napoca, Romania; 11 Department of Pathology, Prof. Dr Ion Chiricuta Oncology Institute, 400015 Cluj-Napoca, Romania; raresbuiga@yahoo.fr; 12 Department of Surgery, Prof. Dr Ion Chiricuta Oncology Institute, 400015 Cluj-Napoca, Romania

**Keywords:** breast cancer, major penetrance gene, pathogenic/likely pathogenic variant, radiomics, machine learning

## Abstract

**Background:** Breast cancer (BC) is the most frequently diagnosed cancer in women and the leading cause of cancer-related deaths in women globally. Carriers of P/LP variants in the *BRCA1*, *BRCA2*, *TP53*, *PTEN*, *CDH1*, *PALB2*, and *STK11* genes have an increased risk of developing BC, which is why more and more guidelines recommend prophylactic mastectomy in this group of patients. Because traditional genetic testing is expensive and can cause delays in patient management, radiomics based on diagnostic imaging could be an alternative. This study aims to evaluate whether ultrasound-based radiomics features can predict P/LP variant status in BC patients. **Methods**: This retrospective study included 88 breast tumors in patients tested with multigene panel tests, including all seven above-mentioned genes. Ultrasound images were acquired prior to any treatment, and the tumoral and peritumoral areas were used to extract radiomics data. The study population was divided into P/LP and non-P/LP variant groups. Radiomics features were analyzed using machine learning models, alone or in combination with clinical features, with the aim of predicting the genetic status of BC patients. **Results**: We observed significant differences in radiomics features between P/LP- and non-P/LP-variant-driven tumors. The developed radiomics model achieved a maximum mean accuracy of 85.7% in identifying P/LP variant carriers. Including features from the peritumoral area yielded the same maximum accuracy. **Conclusions**: Radiomics models based on ultrasound images of breast tumors may provide a promising alternative for predicting P/LP variant status in BC patients. This approach could reduce dependence on costly genetic testing and expedite the diagnostic process. However, further validation in larger and more diverse populations is needed.

## Simple Summary

The latest European and American guidelines (NCCN, ASCO, ESMO), provide recommendations for prophylactic mastectomies or risk-reducing mastectomies to individuals at high risk of breast cancer due to specific genetic pathogenic/likely pathogenic (P/LP) variants. Individualized decision-making should be based on genetic testing results, personal and family cancer history, and personal preferences. Based on current knowledge, breast cancer genetic risk is chiefly defined by P/LP in seven major penetrance genes: *BRCA1*, *BRCA2*, *TP53*, *PTEN*, *CDH1*, *PALB2*, and *STK11*. However, access to genetic testing for these genes varies widely around the world and is often dependent on the country’s healthcare system, reimbursement policies, and the availability of specialized genetic services. In low- and middle-income countries, *BRCA1/2* testing is more commonly available, while in high-income countries all seven genes are tested, making it difficult to apply the guidelines’ recommendations worldwide. Radiomics combined with machine learning algorithms may be a potential cost-effective and time-saving alternative for identifying genetic P/LP variants in breast cancer patients. This study explores the use of radiomics-based analyses of ultrasound images to predict P/LP variant status in breast cancer patients. The development of such a model could revolutionize personalized medicine by enabling faster and more accessible diagnostics.

## 1. Introduction

Breast cancer (BC) is the leading cause of cancer deaths in women worldwide, posing a major public health concern [1,2]. Up to 10% of cases are due to inherited genetic changes, known as hereditary BC. Depending on their lifetime risk of developing BC, genes are classified as high—(>50–80%), moderate—(20–50%), and low-penetrance susceptibility genes [3,4,5,6,7,8]. High penetrance means that individuals carrying a mutation in these genes have a very high risk of developing BC, while moderate penetrance increases BC risk, but not as drastically as high-penetrance genes. Besides penetrance, gene mutations are further classified, based on the strength of evidence that is available so far, as follows: pathogenic, likely pathogenic, variants of uncertain significance, likely benign, and benign [9]. Pathogenic and likely pathogenic genetic mutations have been strongly associated with increased BC risk.

The latest American and European guidelines consider high-risk individuals as those with pathogenic and likely pathogenic mutations in high- or moderate-penetrance genes. For the high-penetrance genes *BRCA1*, *BRCA2*, *TP53*, *PTEN*, and *CDH1*, where BC risk is particularly high, prophylactic mastectomy is a frequently recommended option by the NCCN, ASCO, and ESMO [10,11,12,13]. In cases of *PALB2* and *STK11* high-penetrance genes, prophylactic mastectomy may be an option, but the decision is more individualized, often depending on personal and family history (Table 1).

However, diagnosing all these genes through genetic testing presents several challenges, including high costs and limited accessibility [14]. In addition, the waiting time for genetic test results varies widely, averaging between 4 and 6 weeks, further delaying the treatment process for many patients. Up to 23.2% of patients who were tested before surgery received the results after surgery, necessitating re-interventions, with major implications on quality of care and costs [15]. In light of these limitations, alternative diagnostic methods that are more accessible and cost-effective are needed. Radiomics, an emerging field that extracts quantitative features from medical images through algorithms, is one such alternative. By analyzing images, radiomics can reveal characteristics that are otherwise indiscernible to the human eye [16]. Few studies in the literature have reported the differences in imaging appearances of *BRCA*-positive and non-BRCA tumors and analyzed radiomics’ power to predict genetic status [17,18,19]. However, all of these studies focused only on *BRCA* 1 and 2 genes and did not include multigene panel tests. In the era of personalized medicine, relying on limited genetic information is insufficient. To minimize the need for re-interventions, it is crucial to assess the genetic profile for all seven major penetrance genes. Furthermore, while one study has explored the use of MRI-based radiomics data for this purpose, such technologies remain costly and not widely accessible [20]. In contrast, alongside mammography, breast and axillary ultrasound is part of the minimum recommended standard for breast cancer diagnosis, offering a cost-effective and readily available diagnostic tool.

The aim of our study is to evaluate the potential of radiomic data extracted from pre-treatment ultrasound images in patients with confirmed breast cancer who have undergone multigene panel testing to predict mutational status relevant to prophylactic mastectomy recommendations. The analysis includes not only *BRCA1* and *BRCA2,* but also *TP53*, *PTEN*, *CDH1*, *PALB2*, and *STK11* genes. If successful, radiomics could serve as a complementary or alternative diagnostic tool for genetic testing, offering a faster and more accessible approach for clinical decision-making.

## 2. Materials and Methods

### 2.1. Study Design and Ethical Approval

This retrospective study was conducted at the Institute of Oncology “Prof. Dr. Ion Chiricuță”, and it received approval from the institutional ethics committee (Nr. crt. 131/2024). The ethics committee waived the need for written consent. This study included a cohort of 240 BC patients diagnosed at our institution between 2021 and 2023.

### 2.2. Patient Selection

**Figure 1:**
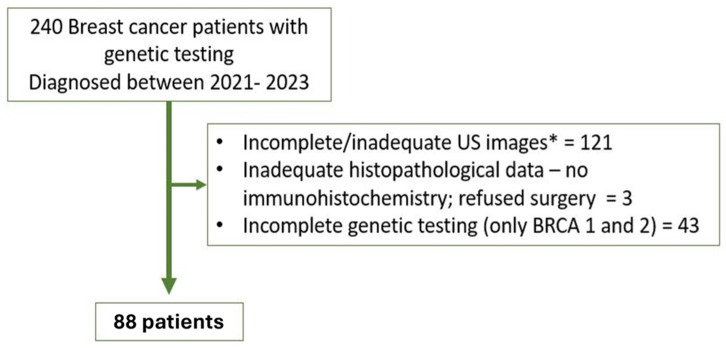
Study population. * US images with measurement, Doppler or elastography.

Patients were consecutively included based on predefined inclusion criteria. Eligible patients were those with confirmed breast cancer, with preoperative breast ultrasound prior to any treatment, tested with a multigene panel test (including *BRCA1* and *BRCA2*, but also *TP53*, *PTEN*, *CDH1*, *PALB2*, and *STK11*), complete surgical intervention, and histopathological analysis. Exclusion criteria included incomplete ultrasound images, inadequate histopathological data, or incomplete genetic testing (only *BRCA* 1 and 2) (Figure 1).

### 2.3. Ultrasound Imaging and Data Acquisition

Breast ultrasound imaging was performed using two systems, GE LOGIQ S7 Expert and SAMSUNG RS85, with a linear probe (5–18 MHz). Only grayscale images were retrieved and interpreted, according to the latest BIRADS lexicon [21], by a dedicated breast radiologist (RP) with 4 year experience. All the segmentations were reviewed by a breast radiologist with over 25 years of experience (CL), and corrections were made before further analysis. For patients with multiple lesions, the most suspicious one was included in the analysis. All images were manually segmented using the Mazda software, focusing on two regions of interest (ROIs): first, the tumor area, represented by the core tumor mass, and second, the peritumoral area, considered as between 0.5 and 1 cm of tissue surrounding the tumor, segmented together with the tumor mass. Posterior enhancement or shadowing was included in the segmentation; the pectoral muscle and the skin were excluded from the area.

### 2.4. Genetics Analysis

After pre-test genetic counselling, eligible patients underwent multigene panel tests with blood or saliva samples using the NGS (next-generation sequencing) technique, enabling the analysis of both SNVs (single-nucleotide variants) and CNVs (copy number variants). In order to select eligible patients, genetic testing criteria as described by the NCCN guidelines were used (Genetic/Familial High-Risk Assessment: Breast, Ovarian, and Pancreatic version 1.2021, and its updated versions which followed subsequently). All of the tests were performed in two external commercial genetic diagnostic laboratories. Patients diagnosed through August 2022 were tested at Invitae, using a customized panel derived from their Multi-Cancer Panel, comprising 84 genes at that time (Invitae, San Francisco, CA, USA). Patients diagnosed from September 2022 onwards were tested at Blueprint Genetics, using a customized panel derived from their Comprehensive Hereditary Cancer Panel, comprising 160 genes (Blueprint Genetics, Keilaranta, Finland). Of note, both panels used for genetic testing included not only the 7 major-penetrance BC genes, but also moderate-penetrance BC genes, and many other genes involved in various other forms of hereditary cancers.

### 2.5. Radiomics Feature Extraction

**Figure 2:**
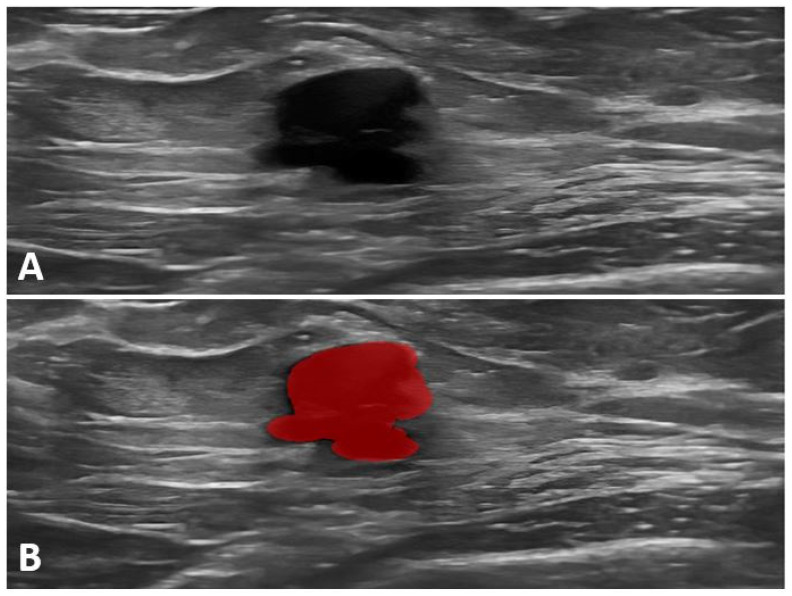
*BRCA1*-positive patient. (**A**) Ultrasound image showing a hypoechoic, irregularly shaped mass with a partially microlobulated and indistinct margin. (**B**) Ultrasound image with segmented tumor area highlighted in red.

Prior to feature extraction, all images were pre-processed using VanceAI Denoise software to reduce noise and normalize image intensity, according to the recommended radiomics guidelines [22]. Radiomics features were extracted using Mazda software after manual segmentation of the ultrasound images. Data were extracted from the two ROIs (1—tumor, 2—tumor + peritumoral area), resulting in two datasets with 306 variables each. The extracted radiomics features included traditional characteristics, such as intensity, texture, and shape, as well as features generated through deep learning algorithms (Figure 2).

### 2.6. Feature Selection, Statistical Analysis, and Model Development

In order to build the model, we used a three-step feature selection process to develop the prediction model: 1—Univariate Analysis: The Mann–Whitney U test was employed to identify features with significant differences between P/LP and non-P/LP BC tumors. The Benjamini–Hochberg method was used for multiple testing correction, and features with adjusted *p*-values less than 0.050 were considered significant. 2—Spearman Correlation: Features with a Spearman correlation coefficient >0.9 or <−0.9 were removed to reduce redundancy. 3—LASSO Regression: A binary logistic regression model with least absolute shrinkage and selection operator (LASSO) was used to select radiomics features, which were then combined into radiomics scores (Rad-Score). All the analyses that were performed for feature selection and described above were performed on data from the full cohort of patients.

The model’s performance was evaluated using multiple machine learning classifiers, and the results were ranked in terms of ROC (Receiver Operating Characteristic) curve analysis, sensitivity, specificity, accuracy, negative predictive value (NPV), and positive predictive value (PPV). The machine learning classifiers were trained and validated using 75% of the data and tested on the remaining 25%, following the current literature recommendations relevant to our study [23].

All analyses were performed using R Statistical Software version 4.4.2 [24].

## 3. Results

### 3.1. Population Characteristics

The final cohort comprised 88 breast tumors; 56.81% (50) patients were positive for at least one mutation and included in the pathogenic-positive group, while 43.18% (38) were negative for all mutations and included in the non-pathogenic group. The median age was 45 years in the pathogenic-positive group and 46 years in the non-pathogenic group. No statistically significant differences in age or histology type of cancer were found between the two groups (Table 2).

We observed a statistically significant difference in the Ki67% proliferation index between the two groups (*p* = 0.005), with pathogenic-positive tumors displaying higher proliferation rates. Other tumor characteristics, including hormonal receptors and HER2 status, did not show significant differences between the two groups (Table 2)**.**

### 3.2. Building the Prediction Model—Training Set

The study group was divided into a 3:1 ratio—75% of the patients were used for training the machine learning classifier algorithms, while the remaining 25% were used for the testing of the model. All analyses were performed separately, first for the tumor only, second for the tumor and peritumoral extracted data.

#### 3.2.1. Feature Selection

**Figure 3:**
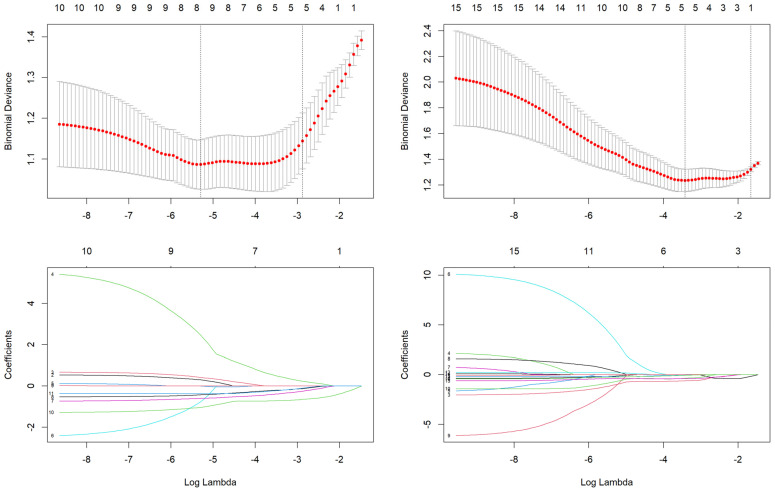
LASSO logistic regression for tumor and peritumoral areas. Upper images: Selection of the tuning parameter lambda (λ) using 10-fold cross validation: left—tumor area, right—peritumoral area. Binomial deviances from the least absolute shrinkage and selection operator regression cross-validation model were plotted as a function of log (λ). Lower images: LASSO coefficient profiles of the 7 and 5 radiomics features with non-zero coefficients, extracted from tumor (**left**) and peritumoral (**right**) data.

A total of 310 features were extracted from the tumor-only and tumor + peritumoral areas for each patient. To exclude a possible batch effect, a *t*-test was applied for the characteristics of the images derived from the two ultrasound machines; the results were not statistically significant (*p*-value > 0.050). To prevent overfitting in the predictive radiomics model, a three-step process was followed for feature selection, aiming to reduce the large number of extracted features to a smaller, more relevant subset. First, a Mann–Whitney U test was conducted to identify individual radiomic features that showed statistically significant differences between the pathogenic-positive and non-pathogenic groups. To account for multiple comparisons, the Benjamini–Hochberg method was applied, adjusting the *p*-values to control the false discovery rate. Features with an adjusted *p*-value of less than 0.050 were considered significant and were retained. This analysis resulted in the identification of 248 significant features for the tumor-extracted data and 88 significant features for the tumor and peritumoral extracted data. Secondly, in order to further address redundancy, we employed a correlation-based feature selection in order to identify highly inter-correlated features (i.e., −0.9 < Spearman coefficient < 0.9). For any two feature pairs that were highly correlated, the feature with the largest mean absolute correlation was removed. This step ensured that only independent, non-redundant features were retained for model construction. This analysis resulted in the identification of 10 significant features for the tumor-extracted data and 15 significant features for the tumor and peritumoral extracted data. The third step involved applying LASSO regression, a method known for its ability to perform both variable selection and regularization to enhance the prediction accuracy of the model. The LASSO model was trained using 10-fold cross-validation, where the data were split into 10 subsets. In each iteration, nine subsets were used for training the model, and the remaining subset was used for validation. This process was repeated until all subsets were used for validation once. After cross-validation, seven radiomic features with non-zero coefficients were selected to build the final model for tumor areas, while five radiomic features with non-zero coefficients were selected to build the final model for tumor + peritumoral areas (Figure 3 and Table 3, Supplemental File S1).

#### 3.2.2. Model Construction and Radiomic Score Calculation

A radiomic score (Rad-Score) was calculated for each patient by combining the seven and five selected radiomic features (Table 3) into a linear combination, weighted by their respective coefficients from the LASSO regression. This Rad-Score represented the likelihood of a patient being pathogenic-positive or non-pathogenic based on their ultrasound imaging features. We use the label Rad-score 1 if the data were extracted only from the tumor, and Rad-score 2 if the data included the tumor and peritumoral area. The Rad-Scores, together with the Ki67% proliferation index, were used as predictors for pathogenic mutation status. The complete code that was generated is publicly available at https://github.com/RP91-web/US-pathogenic-variants/blob/R-code-for-predicting-patogenic-variants/R%20Script%20FLA.txt (accessed on 11 March 2025).

#### 3.2.3. Performance of the Ki67% Proliferation Index and Radiomic Score Derived from Tumor-Only Data (Rad-Score 1)

To evaluate the performance of the predictive model, we employed multiple machine learning algorithms, with Rad-Scores and the Ki67% proliferation index as predictors of pathogenic mutation status. In addition to the classical model accuracy indices, we also computed and generated the corresponding ROC curves.

**Figure 4:**
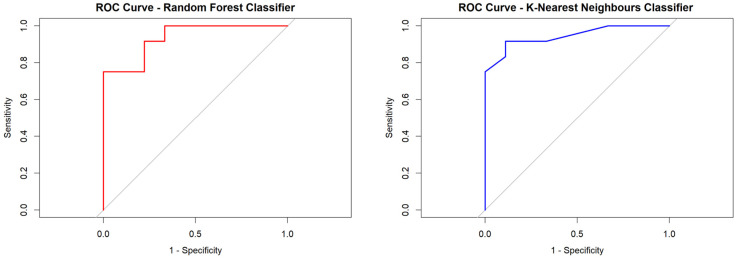
Random Forest and K-Nearest Neighbors classifier-derived ROC curves for predicting pathogenic variants from tumor-only based model.

The best-performing algorithms, with regard to AUC and accuracy, were the Random Forest and K-Nearest Neighbors classifiers, in which Rad-score 1 together with the Ki67% proliferation index predicted pathogenic mutation presence/absence with mean AUCs of 0.935 and 0.939, respectively (Figure 4), a mean accuracy of 80.9%, a mean PPV of 90% (meaning that in 90% of cases the test returned a true positive result), and a mean NPV of 72.7% (meaning that in 72.7% of cases the test returned a true negative result). The mean sensitivity and mean specificity were 75% and 88.8%, respectively (Table 4).

#### 3.2.4. Performance of the Ki67% Proliferation Index and Radiomic Score Derived from Tumor and Peritumoral Data (Rad-Score 2)

**Figure 5:**
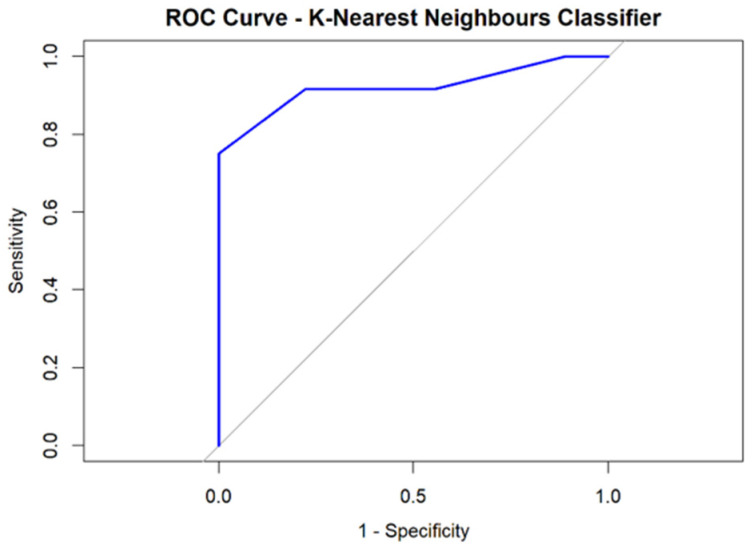
K-Nearest Neighbors classifier-derived ROC curve for predicting pathogenic mutations from tumor and peritumoral data.

The best-performing algorithm, with regard to AUC and accuracy, was represented by the K-Nearest Neighbors classifier, in which Rad-score 2 together with the Ki67% proliferation index predicted pathogenic mutation presence/absence with a mean AUC of 0.930 (Figure 5), a mean accuracy of 80.9%, a mean positive predictive value (PPV) of 78.5% (meaning that in 78.5% of cases the test returned a true positive result), and a mean negative predictive value (NPV) of 85.7% (meaning that in 85.7% of cases the test returned a true negative result). The mean sensitivity and mean specificity were 91.6% and 66.6%, respectively (Table 5).

## 4. Discussion

The current study tested and validated an ultrasound-based radiomics model able to predict the P/LP variant status of breast cancer patients. Higher accuracy was achieved when combining radiomics data extracted from the tumor and peritumoral area, making this a promising alternative to traditional genetic testing.

This could be particularly useful in settings where genetic testing is prohibitively expensive or where patients face long waiting times for results [18,22]. There are studies that have attempted to study imaging differences in genetically tested patients, but these are mainly focused on *BRCA1* and 2 genes [22,25,26,27,28,29]. For the other genes, there are only case reports or small series reported. A single paper based on ultrasound features has shown that patients with P/LP variants in *BRCA*, as well as other major-penetrance genes, such as *PALB2*, may have pseudo-benign features, which may pose diagnostic problems [28].

While radiomics has been widely explored in the context of CT and MRI imaging, its use in ultrasound-based breast cancer diagnosis is relatively novel. Compared to these, ultrasound is much more available and affordable, and in addition is used with all pre-treatment patients to obtain the diagnosis of cancer by performing biopsy. One study predicted *BRCA* status from ultrasound images of the ovaries, obtaining a negative predictive value of 0.73, and accuracy values of 0.72 and 0.79 on a training set [25]. In our study, we used ultrasound images of breast cancer prior to any treatment and obtained comparable results. Moreover, the inclusion of peritumoral features aligns with previous research, which emphasizes the importance of the tumor microenvironment in cancer progression and diagnosis [19,26]. Our findings suggest that radiomics can extract valuable information from both the tumor and the surrounding tissues, leading to improved predictive models. Furthermore, the inclusion of ki67% as a clinical parameter did not improve model performance, suggesting that the radiomics features alone were strong predictors of pathogenic status.

The potential clinical implications of this research are significant. By providing a non-invasive, cost-effective diagnostic tool, radiomics could facilitate earlier and more personalized treatment decisions. Moreover, it may help overcome the limitations of genetic testing by offering faster diagnostic alternatives.

However, despite these promising results, several limitations must be acknowledged. The relatively small sample size limits the generalizability of the findings. Additionally, the use of different ultrasound machines introduces variability in image acquisition, which could affect the reproducibility of the results—but at the same time it is closer to the clinical scenario of clinical life. Although our findings were tested on unseen data (the remaining 25%), the model was not validated on external datasets.

## 5. Conclusions

This study highlights the potential of radiomics as a supplementary tool for predicting P/LP variants in breast cancer patients. By analyzing US images, machine learning models may provide accurate and accessible diagnostics, which could reduce reliance on expensive genetic testing. Future research should focus on larger, more diverse cohorts to validate these findings and further refine imaging-based predictive models.

## Figures and Tables

**Table 1:** Breast cancer susceptibility genes with impact on prophylactic mastectomy.

Gene	Penetrance	Lifetime Breast Cancer Risk %	Prophylactic Mastectomy	Guidelines
*BRCA1*	High	60–87	Recommended	NCCN, ASCO, ESMO, NICE
*BRCA2*	High	45–84	Recommended	NCCN, ASCO, ESMO
*TP53*	High	49–85	Recommended	NCCN, ASCO, ESMO
*PTEN*	High	25–50	Recommended	NCCN, ASCO, ESMO
*CDH1*	High	39–52 *	Recommended	NCCN, ASCO, ESMO
*PALB2*	Moderate	33–58	Suggested/enhanced Surveillance	NCCN
				ASCO
*STK11*	Moderate	32–54	Suggested/enhanced Surveillance	NCCN
				ASCO

* Increased risk of lobular carcinoma; updated NCCN, ASCO, and ESMO guidelines as per ref. nr 10–13.

**Table 2:** Clinical, histology, and immunohistochemistry characteristics of the study group.

	Patients with Pathogenic Variants (N = 50)	Patients Without Pathogenic Variants (N = 38)	*p*-Value
Age (median)	45 (34–65)	46 (34–65)	0.721
Histology ^1^			0.062
IDC-NST	40	24	
ILC	9	7	
DCIS	1	6	
Other	0	1	
Nottingham grade			0.033
0	0	3	
1	2	0	
2	22	23	
3	26	12	
Ki67%			0.005
<20	4	12	
>20	46	26	
ER			0.714
+	31	25	
−	19	13	
PR			0.842
+	20	16	
−	30	22	
HER 2			0.465
+	11	6	
−	39	32	

^1^ IDC-NST—invasive ductal carcinoma of no special type; ILC—invasive lobular carcinoma; DCIS—ductal carcinoma in situ; Other—mixed invasive cancers with mucinous, papillary, medullary features; ER—estrogen receptor; PR—progesterone receptor; HER 2—human epidermal growth factor.

**Table 3:** Final radiomics features that were combined for calculating the Rad-Score 1 and Rad-Score 2 predictors.

Tumor-Only Features (Rad-Score 1)	Coefficient (β)	Feature Description
X.S.0.1.Contrast	0.11	Measures the intensity difference between neighboring pixels, indicating texture roughness or heterogeneity
X.S.2.2.AngScMom	0.94	It quantifies the homogeneity of an image by summing the squared values of the gray-level co-occurrence matrix (GLCM).
X.S.5.5.SumVarnc	−0.01	Represents texture uniformity or energy; higher values indicate more homogenous textures
X135dr_GLevNonU	−0.49	Assesses the distribution of gray levels; lower values indicate more uniform textures
Teta2	−0.29	Represents an angular-related feature in texture analysis, linked to orientation or directional patterns
ZWavEnLL_s6	−0.73	Energy in specific wavelet frequency bands, which may correlate with microstructural variations or subtle changes in tissue composition
ZWavEnLH_s6	−0.32	Energy in specific wavelet frequency bands, which may correlate with microstructural variations or subtle changes in tissue composition
**Tumoral + Peritumoral** **Features (Rad-Score 2)**	**Coefficient (β)**	
Perc.01.	−0.59	First percentile of intensity values, representing the lower bound of pixel intensity distribution
X.S.5.5.SumEntrp	−0.31	Measures randomness in the image texture; higher values indicate more complexity and heterogeneity
Horzl_RLNonUni	−0.03	Evaluates the variability of consecutive pixel runs in the horizontal direction; lower values suggest more uniform textures
WavEnHL_s.3	−0.38	Energy in the high–horizontal and low–vertical frequency wavelet decomposition at scale 3, indicating texture detail at a specific resolution
WavEnHH_s.6	−0.04	Quantify wavelet energy at specific high–low and high–high frequency bands at scales 3 and 6, which may correlate with fine-to-coarse microstructural tissue variations or changes in composition

**Table 4:** Performance of Ki67% and Rad-score 1 as predictors of pathogenic mutation status, as evaluated by multiple machine learning prediction classifiers.

Classifier	AUC	Specificity	Sensitivity	PPV	NPV	Accuracy (95% CI)
Random Forest	0.935	0.888	0.750	0.900	0.727	0.809 (0.580 to 0.945)
Boosting Classification	0.888	0.777	0.916	0.846	0.875	0.857 (0.636 to 0.969)
K-Nearest Neighbors	0.939	0.888	0.750	0.900	0.727	0.809 (0.580 to 0.945
Support Vector Machine	0.851	0.777	0.750	0.818	0.700	0.761 (0.528 to 0.917)
Feature Importance (1-AUC)	Rad-score 1	Ki67%				
Random Forest	0.133	0.297				
Boosting Classification	0.375	0.281				
K-Nearest Neighbors	0.403	0.142				
Support Vector Machine	0.260	0.195				

**Table 5:** Performance of Ki67% and Rad-score 2 as predictors of pathogenic mutation status, as evaluated by multiple machine learning prediction classifiers.

Classifier	AUC	Specificity	Sensitivity	PPV	NPV	Accuracy (95% CI)
Random Forest	0.824	0.666	0.666	0.727	0.600	0.666 (0.430 to 0.854)
Boosting Classification	0.851	0.555	0.916	0.733	0.833	0.761 (0.528 to 0.917)
K-Nearest Neighbors	0.930	0.666	0.916	0.785	0.857	0.809 (0.580 to 0.945)
Support Vector Machine	0.907	0.777	0.916	0.846	0.875	0.857 (0.636 to 0.969)
Feature Importance (1-AUC)	Rad-score 2	Ki67%				
Random Forest	0.292	0.022				
Boosting Classification	0.282	0.152				
K-Nearest Neighbors	0.388	0.164				
Support Vector Machine	0.224	0.102				

## Data Availability

Data supporting the findings of this study are available upon reasonable request from the corresponding author.

## Supplementary Materials

The following supporting information can be downloaded at: https://www.mdpi.com/article/10.3390/cancers17061019/s1, File S1. Confusion matrices for each of the machine learning models.

## References

[1] European Commission (2024). European Cancer Information System Breast Cancer in the E.U..

[2] The International Agency for Research on Cancer (IARC) (2022). Global Cancer Observatory. https://gco.iarc.fr/en.

[3] Filippini S.E., Vega A. (2013). Breast cancer genes: Beyond BRCA1 and BRCA2. Front. Biosci. (Landmark Ed.).

[4] Turchiano A., Piglionica M., Martino S., Bagnulo R., Garganese A., De Luisi A., Chirulli S., Iacoviello M., Stasi M., Tabaku O. (2023). Impact of High-to-Moderate Penetrance Genes on Genetic Testing: Looking over Breast Cancer. Genes.

[5] Weitzel J.N., Neuhausen S.L., Adamson A., Tao S., Ricker C., Maoz A., Rosenblatt M., Nehoray B., Sand S., Steele L. (2019). Pathogenic and likely pathogenic variants in PALB2, CHEK2, and other known breast cancer susceptibility genes among 1054 BRCA-negative Hispanics with breast cancer. Cancer.

[6] Han S.A., Kim S.W. (2021). BRCA and Breast Cancer-Related High-Penetrance Genes. Adv. Exp. Med. Biol..

[7] Daly M.B., Pal T., Berry M.P., Buys S.S., Dickson P., Domchek S.M., Elkhanany A., Friedman S., Goggins M., Hutton M.L. (2021). Genetic/Familial High-Risk Assessment: Breast, Ovarian, and Pancreatic, Version 2.2021, NCCN Clinical Practice Guidelines in Oncology. J. Natl. Compr. Cancer Netw..

[8] Hansford S., Kaurah P., Li-Chang H., Woo M., Senz J., Pinheiro H., Schrader K.A., Schaeffer D.F., Shumansky K., Zogopoulos G. (2015). Hereditary Diffuse Gastric Cancer Syndrome: CDH1 Mutations and Beyond. JAMA Oncol..

[9] Offit K., Couch F.J., Nathanson K.L. (2016). Evaluation of ACMG-Guideline-Based Variant Classification of Cancer Susceptibility and Non-Cancer-Associated Genes in Families Affected by Breast Cancer. Am. J. Hum. Genet..

[10] NCCN Guidelines for Genetic/Familial High-Risk Assessment: Breast, Ovarian, and Pancreatic. https://www.nccn.org/guidelines/guidelines-detail?category=2&id=1503.

[11] ASCO Guidelines for Management of Hereditary Breast Cancer: American Society of Clinical Oncology, American Society for Radiation Oncology, and Society of Surgical Oncology Guideline. https://ascopubs.org/doi/10.1200/JCO.20.00299.

[12] ESMO Guidelines for Risk Reduction and Screening of Cancer in Hereditary Breast-Ovarian Cancer Syndromes: ESMO Clinical Practice Guideline. https://www.esmo.org/guidelines/guidelines-by-topic/hereditary-syndromes/risk-reduction-screening-hereditary-breast-ovarian-cancer-syndromes.

[13] Menko F.H., Monkhorst K., Hogervorst F.B.L., Rosenberg E.H., Adank M.A., Ruijs M.W.G., Bleiker E.M.A., Sonke G.S., Russell N.S., Oldenburg H.S.A. (2022). Challenges in breast cancer genetic testing. A call for novel forms of multidisciplinary care and long-term evaluation. Crit. Rev. Oncol..

[14] Lee S., Rajeev P., Finning S., Oh C., Pothuri B. (2022). Factors associated with delayed genetic testing for patients with BRCA-related cancers (428). Gynecol. Oncol..

[15] Armstrong J., Lynch K., Virgo K.S., Schwartz M.D., Friedman S., Dean M., Andrews J.E., Bourquardez Clark E., Clasen J., Conaty J. (2021). Utilization, Timing, and Outcomes of BRCA Genetic Testing Among Women with Newly Diagnosed Breast Cancer from a National Commercially Insured Population: The ABOARD Study. JCO Oncol. Pr..

[16] Kocak B., Baessler B., Cuocolo R., Mercaldo N., Pinto Dos Santos D. (2023). Trends and statistics of artificial intelligence and radiomics research in Radiology, Nuclear Medicine, and Medical Imaging: Bibliometric analysis. Eur. Radiol..

[17] Pintican R., Duma M.M., Szep M., Feier D., Eniu D., Goidescu I., Chiorean A. (2021). The Role of US in Depicting Axillary Metastasis in High-Risk Breast Cancer Patients. J. Pers. Med..

[18] Lee M.V., Katabathina V.S., Bowerson M.L., Mityul M.I., Shetty A.S., Elsayes K.M., Balachandran A., Bhosale P.R., McCullough A.E., Menias C.O. (2017). BRCA-associated Cancers: Role of Imaging in Screening, Diagnosis, and Management. RadioGraphics.

[19] Deng T., Liang J., Yan C., Ni M., Xiang H., Li C., Ou J., Lin Q., Liu L., Tang G. (2024). Development and validation of ultrasound-based radiomics model to predict germline BRCA mutations in patients with breast cancer. Cancer Imaging.

[20] Vasileiou G., Costa M.J., Long C., Wetzler I.R., Hoyer J., Kraus C., Popp B., Emons J., Wunderle M., Wenkel E. (2020). Breast MRI texture analysis for prediction of BRCA-associated genetic risk. BMC Med. Imaging.

[21] D’Orsi C.J., Sickles E.A., Mendelson E.B., Morris A., Creech E.W., Butler F.P., Wiegmann P.G., Chatfield B.M., Meyer W.L., Wilcox A.P. (2013). ACR BI-RADS Atlas, Breast Imaging Reporting and Data System.

[22] Zhang W., Guo Y., Jin Q. (2023). Radiomics and Its Feature Selection: A Review. Symmetry.

[23] Joseph V.R. (2022). Optimal ratio for data splitting. Stat. Anal. Data Min..

[24] R Core Team (2024). R: A Language and Environment for Statistical Computing.

[25] Nero C., Ciccarone F., Boldrini L., Lenkowicz J., Paris I., Capoluongo E.D., Testa A.C., Fagotti A., Valentini V., Scambia G. (2020). Germline BRCA 1-2 status prediction through ovarian ultrasound images radiogenomics: A hypothesis generating study (PROBE study). Sci. Rep..

[26] Gallivanone F., Bertoli G., Porro D. (2022). Radiogenomics Breast Cancer Diagnosis and Characterization: Current Status and Future Directions. Methods Protoc..

[27] Pintican R.M., Chiorean A., Duma M., Feier D., Szep M., Eniu D., Goidescu I., Dudea S. (2022). Are Mutation Carrier Patients Different from Non-Carrier Patients? Genetic, Pathology, and US Features of Patients with Breast Cancer. Cancers.

[28] Owens D.K., Davidson K.W., Krist A.H., Barry M.J., Cabana M., Caughey A.B., Doubeni C.A., Epling J.W., Kubik M., Landefeld C.S. (2019). Risk Assessment, Genetic Counseling, and Genetic Testing for BRCA-Related Cancer. JAMA.

[29] Braman N., Prasanna P., Whitney J., Singh S., Beig N., Etesami M., Bates D.D.B., Gallagher K., Bloch B.N., Vulchi M. (2019). Association of Peritumoral Radiomics with Tumor Biology and Pathologic Response to Preoperative Targeted Therapy for HER2 (ERBB2)–Positive Breast Cancer. JAMA Netw. Open.

