# Supplementary material for: Predicting Pathogenic Variants of Breast Cancer Using Ultrasound-Derived Machine Learning Models
Source: Cancers (Basel). 2025 Mar 18;17(6):1019. doi: 10.3390/cancers17061019 (PMC11940624; doi:10.3390/cancers17061019)
Supplement: Supplementary file 1 [file cancers-17-01019-s001.zip › cancers-3505083-supplementary.pdf]

Supplementary Materials File S1. Confusion matrices for each of the machine learning models

Tumoral data

Random Forest

		Predicted	
		0	1
Observed	0	8	3
	1	1	9

K-Nearest Neighbours

		Predicted	
		0	1
Observed	0	8	3
	1	1	9

Support Vector Machine

		Predicted	
		0	1
Observed	0	7	3
	1	2	9

Boosting Classification

		Predicted	
		0	1
Observed	0	7	1
	1	2	11

Tumoral and Peritumoral data

Random Forest

		Predicted	
		0	1
Observed	0	6	4
	1	3	8

K-Nearest Neighbours

		Predicted	
		0	1
Observed	0	6	1
	1	3	11

Support Vector Machine

		Predicted	
		0	1
Observed	0	7	1
	1	2	11

Boosting Classification

		Predicted	
		0	1
Observed	0	5	1
	1	4	11
